# Antioxidant, Alpha‐Amylase Inhibitory and Hypoglycemic Activity of *Smallanthus sonchifolius* Leaves from Nepal: An Integrated In Vitro, In Vivo, and In Silico Approach

**DOI:** 10.1002/fsn3.4672

**Published:** 2024-12-26

**Authors:** Prakash Rawal, Bipindra Pandey, Ram Kishor Yadav, Sushil Panta

**Affiliations:** ^1^ School of Health and Allied Sciences Pokhara University Pokhara Nepal; ^2^ Department of Pharmacy Madan Bhandari Academy of Health Sciences Hetauda Nepal

**Keywords:** alpha‐amylase, antioxidant activity, hypoglycemic activity, molecular docking, *Smallanthus sonchifolius*

## Abstract

In 2019, diabetes mellitus affected 9.3% of the global population and accounted for one in nine adult deaths. Plant‐based antioxidants neutralize harmful free radicals, mitigate oxidative stress, and significantly prevent diabetes and its complications. This study evaluated the in vitro antioxidant, alpha‐amylase inhibitory, in vivo oral hypoglycemic, and *in silico* antidiabetic potential of *Smallanthus sonchifolius* (*S. sonchifolius*) leaf extract. Mice (*n* = 25) were divided into five groups after a 16‐h fast with access to water. The groups received distilled water (normal control), metformin (100 mg/kg, standard), or *S. sonchifolius* ethanolic extract at 100, 250, or 500 mg/kg to compare the antidiabetic potential of the extract with that of the control. Fasting blood glucose levels were measured in tail vein blood before the experiment and at 30, 60, and 120 min post‐administration to evaluate the hypoglycemic activity of the extract and standard drug. The ethanolic extract exhibited dose‐dependent alpha‐amylase inhibition (IC_50_ value 0.136 mg/mL) and significant hypoglycemic effects, reducing blood glucose levels similar to those of the standard drugs voglibose and metformin. The maximum blood glucose reduction was 17.99% and 15.74% in the normal and glucose‐loaded mice, respectively, at 500 mg/kg within 120 min. *In silico* analysis shows, polymatin B and chlorogenic acid demonstrating the highest binding affinity of −8.2 and −7.2 kcal/mol, respectively, in PPAR‐γ (3G9E). Polymatin B and chlorogenic acid showed strong binding affinities of −7.3 and −7.5 kcal/mol, respectively, in alpha‐amylase (4W93). These findings indicate that *S. sonchifolius* possesses significant hypoglycemic and antioxidant properties, suggesting its potential as an antidiabetic agent, warranting further clinical research.

## Graphical abstract

*Smallanthus sonchifolius* extracts show promising antidiabetic potential, with water extract exhibiting high antioxidant activity and ethanol extract demonstrating dose‐dependent alpha‐amylase inhibition and hypoglycemic effects. Polymatin B and chlorogenic acid revealed strong binding affinities in silico studies.

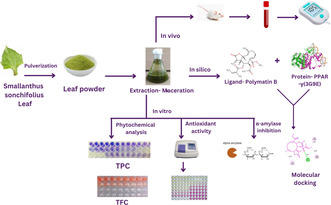

**Abbreviations**
DM: diabetes mellitus
DPPH: 2,2‐diphenyl‐1‐picrylhydrazyl
GAE: gallic acid equivalent
OECD: Organization for Economic Cooperation and Development
OGTT: oral glucose tolerance test
PPAR‐**γ**
: peroxisome proliferator activated receptor gamma
QE: quercetin equivalent
SEM: standard error of mean; *S. sonchifolius*, *Smallanthus sonchifolius*

## 1 Introduction

Diabetes mellitus (DM) is a chronic endocrine metabolic disorder that caused 9.3% of the world population to bear sore torture in 2019. Approximately 11% of mortality among adults age range 20–79 years is due to diabetes mellitus (Atlas D. 2019; Saeedi et al. 2020). DM is categorized into three types based on clinical manifestations: insulin‐dependent (T1DM), insulin‐resistant (T2DM), and gestational DM (Li et al. 2017). In diabetes, hyperglycemia may be caused either by inadequate insulin secretion (T1DM), development of insulin‐resistant tissues (T2DM), or combined conditions (Uddin et al. 2014). Ultimately, as a result of these, severe complications are produced in T1DM and T2DM such as nephropathy, retinopathy, neuropathy, and cardiovascular diseases (Aidi Wannes and Marzouk 2016). Eating healthy diet and regular physical exercise are nonpharmacological approaches to manage diabetes whereas insulin therapy in addition to oral antidiabetic agent of classes sulfonylureas and biguanides are commonly used pharmacological agent (Tafesse et al. 2017). Numerous traditional medicinal plants like *Momordica charantia* , *Allium sativum* , *Azadirachta indica* , *Trigonella foenum*, *Vinca rosea* , and *Ocimum sanctum* are reported to have hypoglycemic properties (Semwal et al. 2009). Currently, existing oral hypoglycemic agents have side effects, such as hypoglycemia, anemia, and cholestatic jaundice (Dchimmer 1996). Natural product‐based medicine has shown beneficial effects in diabetes treatment; however, scientific evidence concerning the efficacy and mechanism of action of this therapy is often indistinct (Semwal et al. 2009).

Some studies have reported that the generation of reactive oxygen species (ROS) causes chronic hyperglycemia, which is related to the pathogenesis of kidney damage in diabetic patients (Dembinska‐Kiec et al. 2008). Hence, this complication may be prevented through ROS scavenging via a free radical scavenging mechanism. Most bioactivephytoconstituents, such as phenolic, alkaloid, and flavonoid compounds, cure endogenous cells and cellular proteins through their free radical scavenging activity (Zhang et al. 2015). Such preventive effects are important for diabetic diseases, which are also due to oxidative processes. Furthermore, phenolic compounds exhibit antidiabetic properties through different mechanisms, such as enhanced insulin release, reduced lipid–glucose absorption in the intestine, and pancreatic beta cell protection caused by cytokine‐induced toxicity (Zhang et al. 2015). Therefore, scientific studies on medicinal herbs are crucial for the development and formulation of novel and natural antidiabetic agents.

*Smallanthus sonchifolius* (*S. sonchifolius*), belonging to family Asteraceae, commonly called yacon in Nepali language (in English; ground apple), is an edible tuberous plant indigenous to the Andes (Fernández et al. 2006). In yacon leaf, there is presence of high level of antioxidant type compound (Lachman, Fernández, and Orsák 2003). It shows diverse pharmacological activities comprising leucocytes' movement restriction (Schorr and Da Costa 2005), immune response modification (Lebeda et al. 2003), cytoprotection effects (Valentová, Sersen, and Ulrichová 2005), and antimicrobial activity (Lin, Hasegawa, and Kodama 2003).

Exploration of bioactive phytoconstituents from natural sources is always a choice for finding novel drugs. The study of *S. sonchifolius* leaf may enhance the proper use of such plants in several disease conditions as an alternative treatment regimen and helps to identify possible therapeutic agents for specific diseases. In the present study, we aimed to explore the in vitro antioxidant and alpha‐amylase activities, in vivo hypoglycemic activity, and phytochemical profiles of *S. sonchifolius* leaf extract using molecular docking. Furthermore, the interaction of ligands with specific receptors was established by *in silico* experiments in favor of significant in vitro and in vivo activities. In addition, we selected the primary phytoconstituents of *S. sonchifolius* leaf as ligands. Likewise, the active site of peroxisome proliferator activated receptor gamma (PPAR‐γ) and the catalytic site of alpha‐amylase were used as target receptors to determine the possible hypoglycemic interactions between phytoconstituents and the enzyme comprising PPAR‐γ and alpha‐amylase. Our research uniquely employs molecular docking analyses to identify interactions between key phytoconstituentswith diabetes management targets like alpha‐amylase and PPAR‐γ, providing a fresh perspective on their hypoglycemic effects at the molecular level with integration with in vitro antidiabetic acitivity and antioxidant activity.

## 2 Experimental Section

### 2.1 Drugs and Chemicals

Ethanol was purchased from Changshu Hongsheng Fine Chemical Co (China). Ltd., while the voglibose tablet was obtained from Quest Pharmaceuticals, Nepal. Starch powder was obtained from Thermo Fisher Scientific India Pvt. (Mumbai, India). Metformin powder was obtained as a reference standard from Summy Pharmaceuticals (Nepal). Alpha‐amylase was purchased from HiMedia (India). The Match glucometer and its strips were obtained from the local scientific market in Pokhara, Nepal. All analytical grade chemicals and reagents were used in this study.

### 2.2 Study Plant Material

*Smallanthus sonchifolius* leaf were collected from Kaski, Nepal. The plant herbarium was authenticated by a taxonomist at the National Herbarium and Plant Laboratories, Kathmandu, Nepal. In addition, a copy of the herbarium (Acc. No. 02‐NPC‐2020) was deposited at the School of Health and Allied Sciences, Pokhara University, Nepal for future reference.

### 2.3 Extraction Procedure

The leaf of *S. sonchifolius* were dried and ground to introduce double maceration at room temperature. Ethanol (99.5% pure) and distilled water were used as extraction solvents with occasional shaking for 3 days. Extraction solvent (i.e., ethanol or water) and *S. sonchifolius* leaf powder in the ratio of 5:1 is used for the maceration process. The content was then passed through Whatman No. 1 filter paper to obtain the crude liquid extract. Afterward, it was concentrated using a rotary evaporator under reduced pressure (60 mmHg) at 40°C, followed by complete drying, and finally stored at 4°C. Extraction yield was determined as follows.

$$\%\text{extraction yield}=\left(\text{gram of dried extract}/\text{gram of leaf used for extraction}\right)\times 100$$

### 2.4 Experimental Animals, Housing, and Ethical Statement

Adult Swiss albino male mice weighing 25–40 g were procured from Department of Plant Resources, Kathmandu, Nepal, and then acclimatized to common laboratory setting for 10 days prior to investigation. The mice were given free access to drinking water and a standard pellet diet. Animal housing was maintained at 23°C ± 2°C with alternating light–dark cycles of 12 h every day. Ethical approval (Refs. No. 86/076/077) was assured by the Institutional Review Committee (IRC) of Pokhara University, Nepal, before conducting the animal experiments. Experimental mice were maintained and handled according to the practices detailed by the National Institutes of Health as the Guide for the Care and Use of Laboratory Animals (NIH 2011).

### 2.5 Qualitative Phytochemical Analysis

The water and ethanol *extracts of S. sonchifolius* undergo phytochemical analysis for the detection of plant secondary metabolites, such as alkaloids, saponins, flavonoids, glycosides, tannins, phenols, terpenoids, and carbohydrates (Evans 2002; Yadav et al. 2014).

### 2.6 Quantitative Phytochemical Analysis

Total phenolic content (TPC) in ethanolic and aqueous extracts was measured using the Folin–Ciocalteu reagent (Singleton and Rossi 1965) with some modifications, and the results are represented as milligrams (mg) of gallic acid (GA) equivalents per gram of dry leaf extract (mg GAE/g). Similarly, the total flavonoid content (TFC) was measured using the aluminum chloride method (Arvouet‐Grand et al. 1994) with slight modifications, and the TFC was represented as mg of quercetin (QE) equivalents per gram of dry leaf extract (mg QE/g).

### 2.7 DPPH Free Radical Scavenging Assays

Antioxidant activity based on the DPPH scavenging potency of the *S. sonchifolius*leaf extract was evaluated according to a previously described method (Molyneux 2004). Briefly, 60 μM DPPH methanolic solution was prepared, and 2 mL of the mixture was mixed with an equal volume of extract at various concentration (0.1–100 μg/mL). The cells were incubated for 30 min at 25°C in the dark. Ascorbic acid (AA) was used as the positive control. The absorbance at 517 nm was measured, and a lower absorbance represents a higher scavenging activity (Blois 1958; Shodehinde and Oboh 2013). The IC_50_ value (i.e., the concentration necessary to scavenge 50% of radicals) of the sample containing plant extracts was also measured. Radical scavenging activity of extract was determined as follows.

$$\text{DPPH free radical scavenging activity}\;\left(\%\right)=\left[\left(A_{\text{control}\;517}–A_{\text{Sample}\;517}\right)/A_{\text{control}\;517}\right]\times 100$$

where, *A*_control 517_ is the control absorbance and *A*_sample 517_ is the plant extract sample absorbance.

### 2.8 In Vitro Alpha‐Amylase Inhibitory Activity

The alpha‐amylase inhibitory potency of the extract was determined using starch iodine reagent (Hossain et al. 2008; Uddin et al. 2014). Briefly, 1 mL of alpha‐amylase was mixed with 1 mL of extracts (0.125–2 mg/mL) and incubated for 10 min at set temperature of 37°C. One milliliter of starch solution (1%) was added and incubated at 37°C for 1 h. In order to stop the reaction, 0.05 mL of 1 M HCl was added followed by 0.1 mL iodine solution. Finally, 2 mL of distilled water was added to adjust the absorbance to 565 nm. Voglibose was used as the standard, whereas phosphate buffer was used as the control and treated similarly to the extract. All experiments were performed in triplicates. The IC_50_ value was determined using regression analysis.

$$\%\alpha -\text{amylase inhibition}=\left[\left(A_{0}-A_{1}/A_{0}\right)\times 100\%\right]$$

where, *A*_1_ = absorbance of negative control and *A*_0_ = absorbance of sample.

### 2.9 Acute Toxicity Study of Plant Extract

Adopting a previously reported methodology of OECD Guidelines 423 (OECD 1996), the acute toxicity of the extract was analyzed. Briefly, the animals were administered a fixed dose in two phases. Subsequently, cardinal signs and symptoms of toxicity were observed for 4–72 h. Additionally, the death of animals after dosing represents the lethal dose (LD_50_). Based on the oral acute toxicity studies of *S. sonchifolius* extract upto 5000 mg/kg, we chose the experimental dose based on the one‐tenth dose of this lethal dose that is 500 mg/kg and its lower dose that is 250 mg/kg and 100 mg/kg for hypoglycemic activity.

### 2.10 In Vivo Hypoglycemic Activity

#### 2.10.1 Experimental Design

Initially, 16 h of fasting was achieved in all mice prior to the experiment with water *ad libitum*, and then the mice were randomly divided into five different groups (*n* = 5).

Group I (control): Distilled water (0.5 mL) was orally administered to the mice.

Group II (standard): 100 mg/kg of metformin was orally administered to mice.

Group III–V as test: *S. sonchifolius* ethanolic leaf extract were orally administered at doses of 100, 250, and 500 mg/kg, respectively.

#### 2.10.2 Hypoglycemic Effect and Oral Glucose Tolerance Test (OGTT) in Normal Mice

Blood was collected from the tail vein for the estimation of glucose levels. Blood samples before the extract, drug, or distilled water were used to measure fasting blood glucose levels. Fasting blood glucose levels were obtained at the start of the experiment to establish a baseline for subsequent measurements. Blood samples taken 30, 60, and 120 min after sample administration were used to predict the hypoglycemic activity of the extract and standard drug (Barham and Trinder 1972; Bhat et al. 2005).

Various concentrations of *S. sonchifolius* leaf ethanolic extracts were used to perform the OGTT in non‐diabetic mice, as described previously (Du Vigneaud and Karr 1925; Sellamuthu et al. 2009). Thirty minutes after 30 min of vehicle or drug administration, 2 g/kg glucose solution was administered orally. The tail vein was preferred for blood collection prior to estimating the serum glucose concentration at 0, 30, 60, and 120 min after glucose administration using a glucometer.

### 2.11 Molecular Docking

**FIGURE 1:**
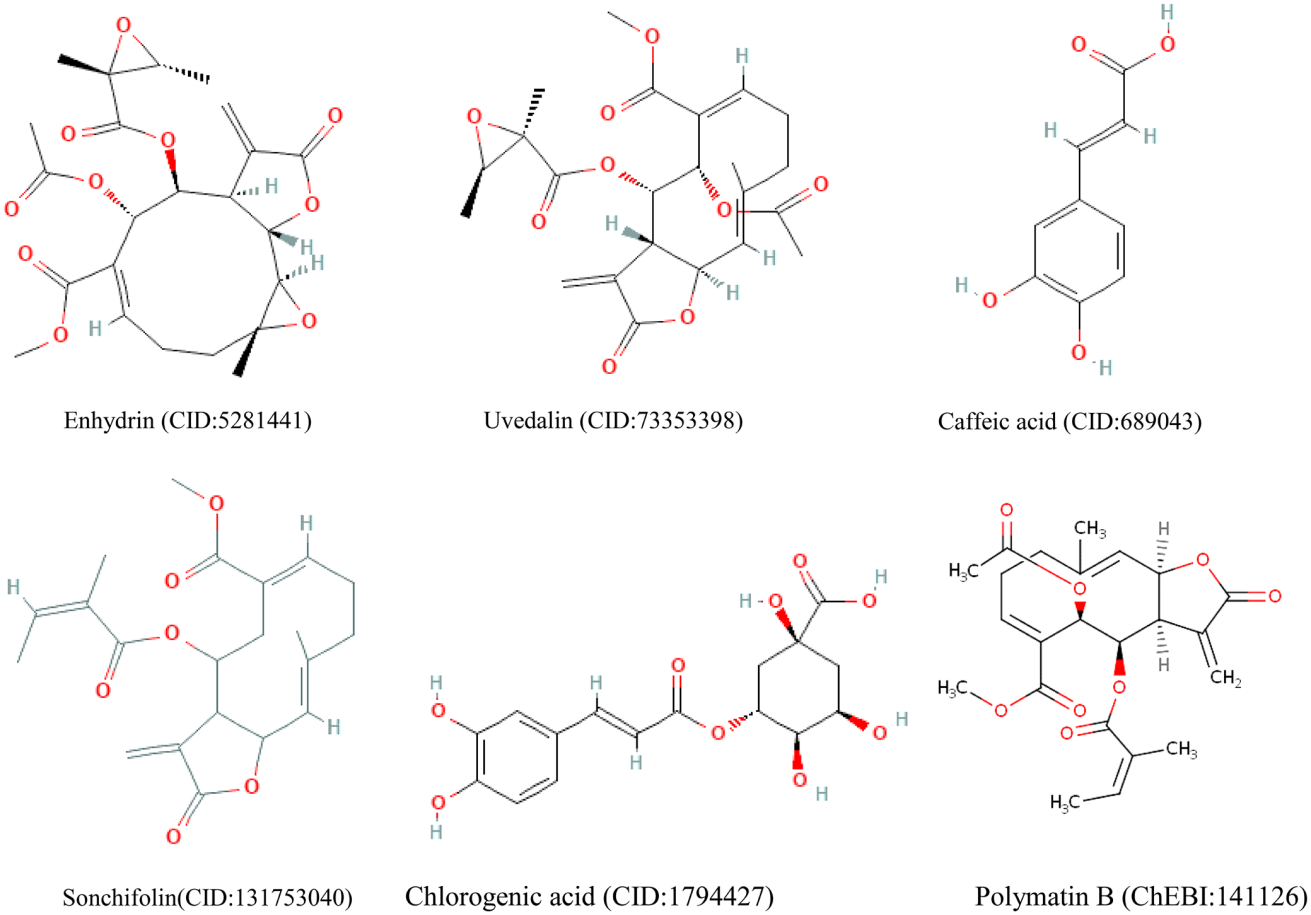
Phytoconstituents from leaves of *Smallanthus sonchifolius* retrieved from PubChem (CID) and Chemical Entities of Biological Interest (ChEBI) database.

Several studies on *S. sonchifolius* have revealed the presence of various phytoconstituents (de Andrade et al. 2017; De Ford et al. 2015). The common isolated constituents like uvedalin, enhydrin, caffeic acid, sonchifolin chlorogenic acid, and polymatin B was retrieved from Google Scholar using “Compound isolated from leaf of *S sonchifolius*” as prompt for molecular docking studies (Figure 1).

The 3D conformers of the phytoconstituents were downloaded from the Structure Data Format (SDF) from the PubChem database (https://pubchem.ncbi.nlm.nih.gov/). Subsequently, the BIOVIA Discovery Studio Visualizer was used to transform the SDF into PDB format. Likewise, 3D crystal structure of target protein: alpha‐amylase (x‐ray diffraction 1.35 Å; 4 W93) (Williams et al. 2015) and peroxisome proliferator‐activated receptor (PPAR‐γ) (x‐ray diffraction 2.3 Å; 3G9E) (Bénardeau et al. 2009) were obtained from Protein Data Bank (PDB) (https://www.rcsb.org/). The selected receptor protein and phytoconstituents were purified by eliminating water and fixing the polar hydrogen and Kollman charges. Finally, the prepared target protein was converted and stored in the PDBQT format using Auto Dock Tools 1.5.7.

**FIGURE 2:**
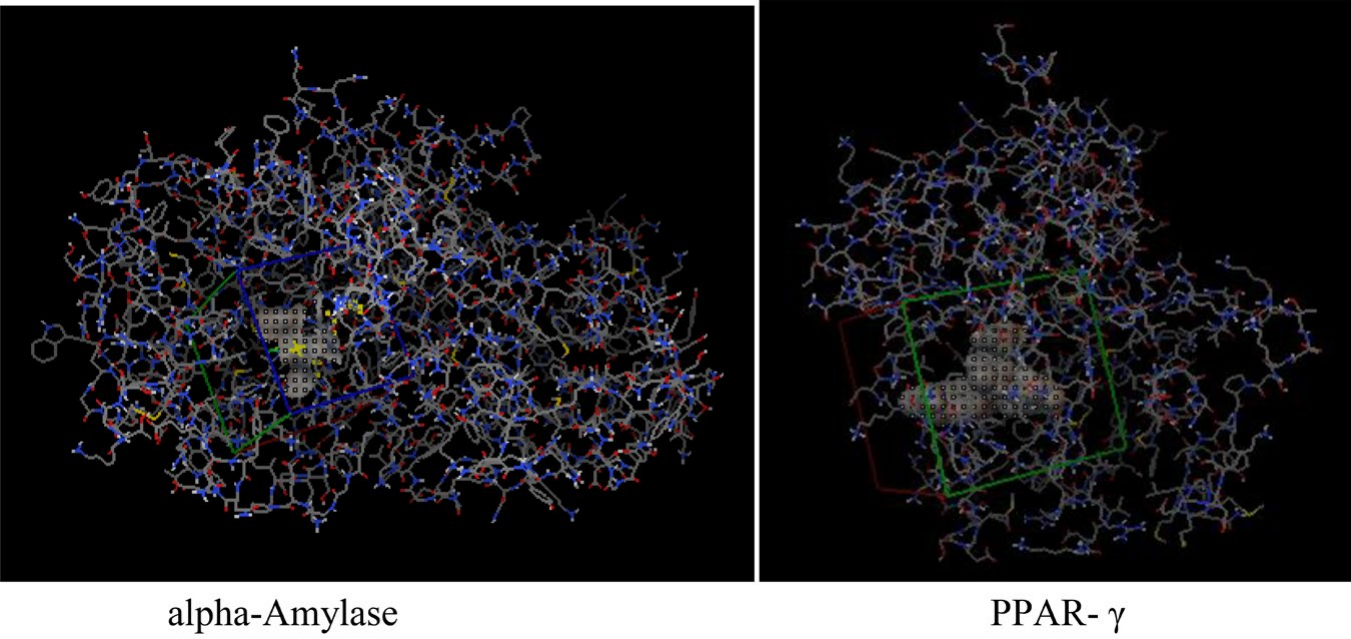
Grid box enclosing the docking site (catalytic site) of target protein alpha‐amylase and PPAR‐γ.

The PDBQT files of target receptors and phytoconstituents were introduced for docking using AutoDock Vina in a space represented by an agrid box of 40 × 40 × 40 sizes with coordinates *x* = −8.186, *y* = 9.463, and *z* = −18.667 for alpha‐amylase, *x* = −4.028; *y* = 31.051; and *z* = 23.177 for PPAR‐γ, with spacings of 0.375 and 0.503 Å, respectively, encompassing catalytic site residues. The most favored binding complex (higher negative value in kcal/mol) was selected and assessed for the amino acid residue and its interaction nature using the BIOVIA Discovery Studio Visualizer 2020 (Figure 2).

### 2.12 Statistical Analysis

All findings are presented as mean ± standard error of mean. A gallic acid and quercetin standard calibration curve was drawn to measure the phenol and flavonoid content using the regression line equation in Microsoft Excel 2007. The findings are presented in the table and figure below. The computational software SPSS version 16 was used for the statistical analysis.

## 3 Results

### 3.1 Qualitative Examination of Phytochemicals

Qualitative estimation of phytochemicals showed the occurrence of various secondary metabolites, such as alkaloids, phenols, flavonoids, carbohydrates, tannins, and a lack of glycosides in both extracts, as tabulated in Table 1.

**TABLE 1:** Phytochemical screening of water and ethanol extracts of *Smallanthus sonchifolius* leaf.

Phytochemical constituents	Specific tests	Water extract	Ethanol extract
Alkaloid	Mayer's test	−	−
	Wagner test	+	+
Carbohydrate	Molish's test	+	+
	Benedict's test	+	+
Glycoside	Modified Borntrager test	−	−
Saponin	Foam test	+	−
Phenol	Ferric chloride test	+	+
Flavonoid	Alkaline reagent test	+	+
Tannin	Gelatin test	−	−
	Ferric chloride test	+	+
Terpenoid	Salkowaski test	+	−
	Copper acetate test	−	+

*Note:* +: presence, −: absence.

### 3.2 Quantitative Examination of Phytochemicals

The extraction yield percentage was the highest for the *S. sonchifolius* water leaf extracts (22.01%). In this study, water extracts of *S. sonchifolius* leaf contained a high concentration of phenol (i.e., 88.46 ± 2.13 mg GAE/g dried weight mass) and flavonoid content (i.e., 424.12 ± 2.86 mg QE/g dry weight mass), as shown in Table 2.

**TABLE 2:** Extraction yield (%) of both solvents of *Smallanthus sonchifolius* leaf and total phenolic and flavonoid contents.

Extract	Extraction yield (%)	Phenols (mg GAE/g dry extract weight)	Flavonoids (mg QE/g dry extract weight)
Water extract	22.01	88.46 ± 1.22	424.12 ± 1.65
Ethanol extract	10.24	70.71 ± 2.43	224.66 ± 1.65

*Note:* Values calculated from the mean of three times experiment and represented as mean ± standard error of mean (*n* = 3).

### 3.3 DPPH Radical Neutralizing Activity

The water extracts of *S. sonchifolius leaf* (IC_50_ value 47.64 μg/mL) showed good activity in scavenging free DPPH radicals, whereas other ethanol extracts showed weak antioxidant activity in comparison with the ascorbic acid standard (Table 3).

**TABLE 3:** DPPH free radical scavenging activity and IC_50_ values of *Smallanthus sonchifolius* leaf extracts at different concentrations.

Extract/Standard	% DPPH scavenging activity				IC_50_ μg/mL
	0.1 μg/mL	1 μg/mL	10 μg/mL	100 μg/mL	
Water extract	10.34 ± 0.74	16.67 ± 0.12	40.15 ± 0.96	82.12 ± 0.42	47.64
Ethanol extract	5.84 ± 0.32	10.95 ± 0.42	26.64 ± 0.56	75.91 ± 0.42	58.86
Ascorbic acid	13.38 ± 0.43	44.28 ± 0.31	95.38 ± 0.12	96.48 ± 0.12	3.56

*Note:* Values calculated from the mean of three times experiment and represented as mean ± standard error of mean (*n* = 3).

### 3.4 In Vitro Inhibitory Activity of Alpha‐Amylase

The ethanol extract of *S. sonchifolius* leaf showed potent inhibitory activity against alpha‐amylase (IC_50_ value 0.136 mg/mL), which is equivalent to that of standard Voglibose, with an IC_50_ value of 0.126 mg/mL in a way contigent on dosage are tabulated in Table 4.

**TABLE 4:** Alpha‐amylase inhibitory activity and IC_50_values of water and ethanol extracts of *Smallanthus sonchifolius* leaf.

Extract/Standard	Concentration (mg/mL)					IC_50_ value (mg/mL)
	0.125	0.25	0.50	1	2	
Water extract	41.03 ± 3.04	56.01 ± 2.14	74.65 ± 0.12	83.31 ± 0.52	91.80 ± 0.17	0.168
Ethanol extract	48.51 ± 1.63	69.47 ± 0.90	83.33 ± 0.08	89.70 ± 0.14	93.62 ± 0.04	0.136
Voglibose	44.58 ± 3.64	70.51 ± 0.69	85.09 ± 0.46	91.06 ± 0.42	94.68 ± 0.17	0.126

*Note:* Mean ± standard error of mean (*n* = 3) calculated from the three times experiment which are represented in above table.

### 3.5 Plant Extracts Acute Toxicity Study

The plant extracts (up to 5000 mg/kg) did not reveal any signs of general toxicity or mortality, as mentioned in the Organization for Economic and Cooperation Development (OECD) guidelines. The ethanol *S. sonchifolius* leaf extract is considered safe for mice.

### 3.6 Acute Hypoglycemic Effects on Normal Mice

**FIGURE 3:**
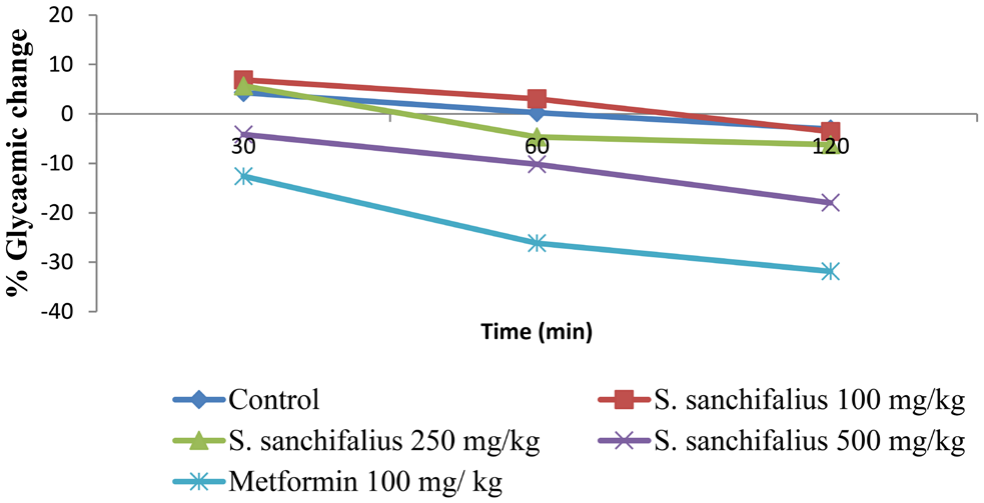
Percentage glycemic change in normal mice.

Three different dosages of *S. sonchifolius* ethanolic extract were tested for their hypoglycemic effect over the course of 16 h in fasting mice at various intervals. The results are presented in Table 5 and Figure 3. The blood glucose levels of the corresponding groups were compared with their initial blood glucose levels (0 min) at various time intervals (30, 60, and 120 min). The findings demonstrated that *S. sonchifolius* displayed a dose‐dependent drop in blood glucose levels over a range of time periods.

**TABLE 5:** Effect of *Smallanthus sonchifolius* ethanolic leaf extract on acute hypoglycemic effects in non‐diabetic mice.

Experimental group (*n* = 5)	Average blood glucose level (mg/dL) at various time intervals (% change in blood glucose level with respect to zero time)			
	0 min	30 min	60 min	120 min
Control	149.20 ± 2.57	155.60 ± 1.53 (4.29%)	149.60 ± 1.50 (0.27%)	144.60 ± 3.07 (−3.08%)
*S. sonchifolius*; 100 mg/kg	162.80 ± 3.72	174.00 ± 6.05 (6.88%)	167.80 ± 5.17 (3.07%)	157.00 ± 4.70 (−3.56%)
*S. sonchifolius*; 250 mg/kg	183.20 ± 3.58	193.60 ± 1.94 (5.68%)	174.60 ± 5.87 (−4.69%)	171.80 ± 5.66 (−6.22%)
*S. sonchifolius*; 500 mg/kg	149.00 ± 5.53	142.80 ± 4.52 (−4.16%)	133.80 ± 5.47 (−10.20%)	122.20 ± 4.59 (−17.99%)
Metformin; 100 mg/kg	171.40 ± 3.02	149.80 ± 2.35 (−12.60%)	126.60 ± 2.75 (−26.14%)	116.80 ± 3.24 (−31.86%)

*Note:* The mean ± standard error of mean (*n* = 5) calculated from the five experiments are presented in the above table. Data in parentheses represent % change in blood glucose level with respect to the corresponding zero time, and a negative sign indicates a decrease in blood glucose level.

*Smallanthus sonchifolius* (500 mg/kg) at 30 min (4.16%), 60 min (10.20%), and 120 min (17.99%); *S. sonchifolius* (250 mg/kg) at 60 min (4.69%) and 120 min (6.22%); and *S. sonchifolius* (100 mg/kg) at 120 min (3.56%) exhibit the significant decrease in blood level which is comparable with the standard drugs metformin (100 mg/kg) evidence of a drop in glucose levels at 30 min (12.60%), 60 min (26.14%), and 120 min (31.86%).

### 3.7 Oral Glucose Tolerance Test

**FIGURE 4:**
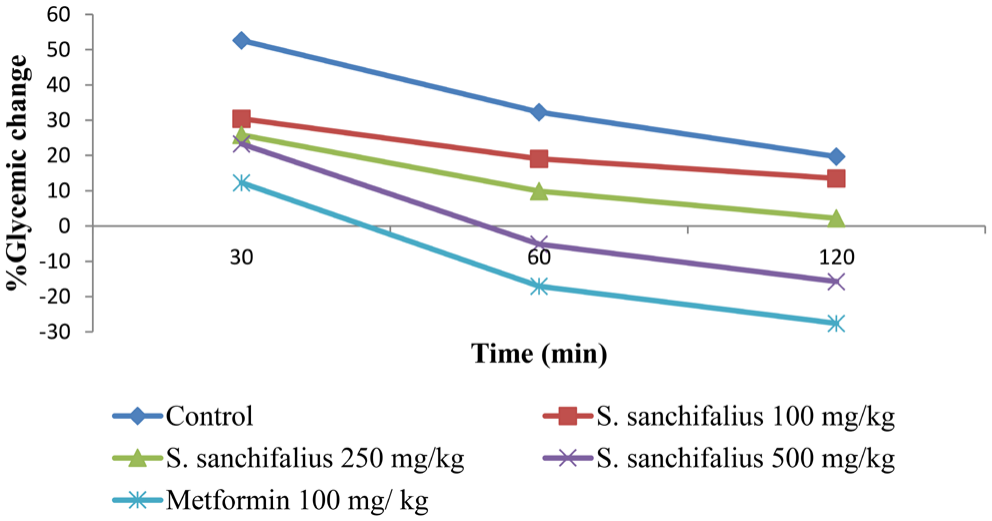
Percentage glycemic change in glucose‐induced hyperglycemic mice.

Oral Glucose Tolerance Test was used to perform the glucose‐induced hyperglycemic test. Mice administered glucose were used to test the hypoglycemic effect of the ethanolic extract of *S. sonchifolius*, and blood glucose levels were measured at various intervals. The findings showed that following oral treatment with glucose at a dose of 2 g/kg, the glucose level in the blood increased in all the groups and decreased gradually. Blood glucose levels were compared for each group's initial blood glucose level (0 min) at various time intervals (30, 60, and 120 min). The results showed that *S. sonchifolius* exhibited a concentration‐dependent decrease in blood glucose levelsat different times. The decrease in blood glucose levels by *S. sonchifolius* (500 mg/kg) at 120 min (15.74%) and 60 min (5.16%), which is equivalent to the standard drug metformin (100 mg/kg), exhibited diminished blood glucose levels at 120 min (17.06%) and 60 min (26.73%) (Table 6 and Figure 4).

**TABLE 6:** Effect of *Smallanthus sonchifolius* ethanolic leaf extract on acute hypoglycemic effects in glucose‐induced hyperglycemic mice.

Experimental group (*n* = 5)	Average blood glucose level (mg/dL) at various time intervals (% change in blood glucose level concerning zero time)			
	0 min	30 min	60 min	120 min
Control	144.44 ± 3.68	220.40 ± 5.33 (52.63%)	191.00 ± 2.95 (32.27%)	172.80 ± 3.69 (19.67%)
*S. sonchifolius*; 100 mg/kg	158.40 ± 3.47	206.60 ± 2.87 (30.43%)	188.60 ± 3.86 (19.07%)	179.80 ± 2.55 (13.51%)
*S. sonchifolius*; 250 mg/kg	155.60 ± 3.26	195.80 ± 3.41 (25.84%)	171.00 ± 3.80 (9.90%)	159.00 ± 3.21 (2.19%)
*S. sonchifolius*; 500 mg/kg	158.80 ± 2.63	195.80 ± 4.16 (23.30%)	150.60 ± 1.91 (−5.16%)	133.80 ± 3.98 (−15.74%)
Metformin; 100 mg/kg	159.40 ± 3.17	179.00 ± 2.88 (12.30%)	132.20 ± 6.01 (−17.06%)	115.40 ± 2.27 (−27.60%)

*Note:* The mean ± standard error of mean (*n* = 5) calculated from the five experiments are presented in the above table. Data in parentheses represent % change in blood glucose level with respect to the corresponding zero time, and a negative sign indicates a decrease in blood glucose level.

### 3.8 Molecular Docking

**FIGURE 5:**
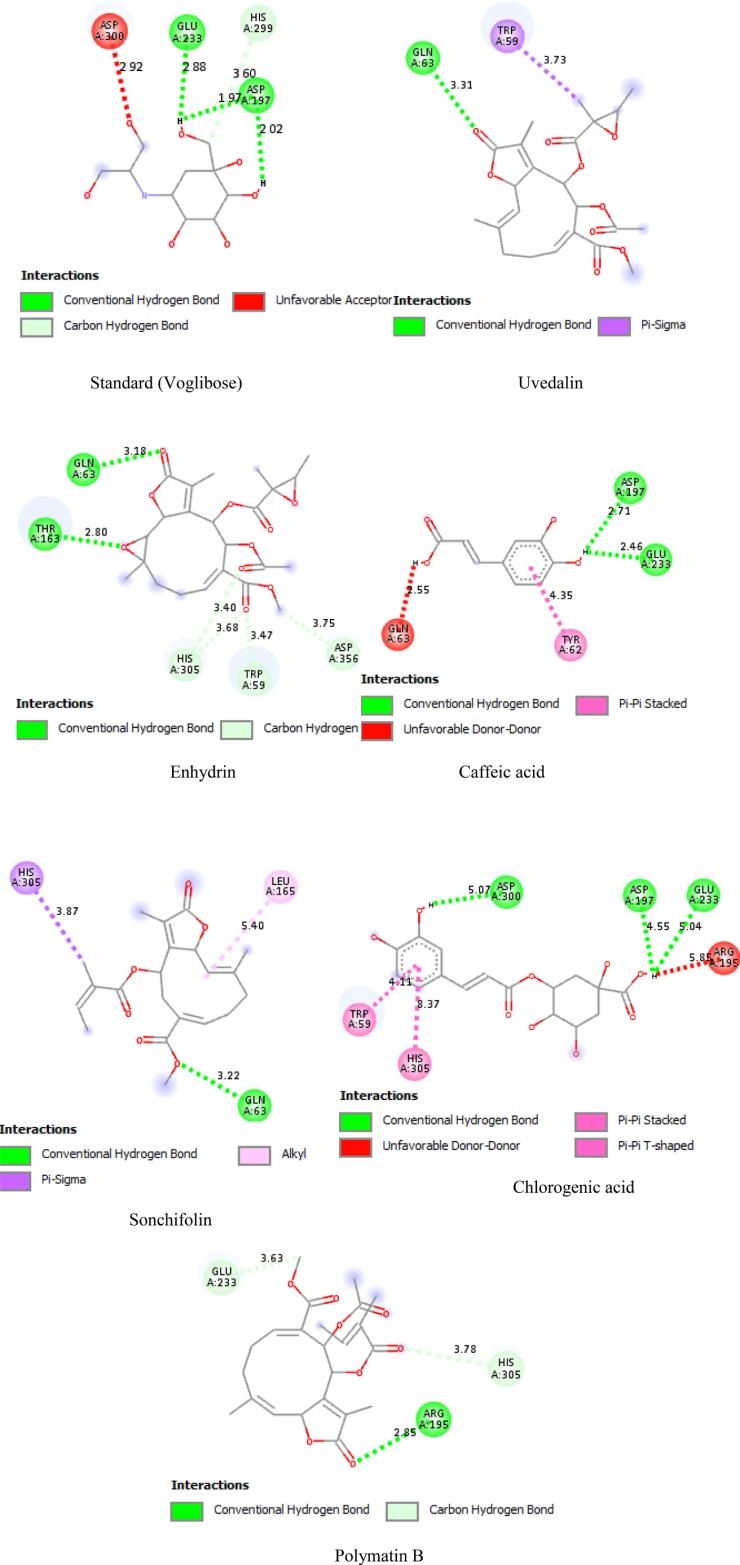
Interaction mode between the alpha‐amylase (4W93) catalytic site and phytoconstituents from leaves of *Smallanthus sonchifolius*.

**FIGURE 6:**
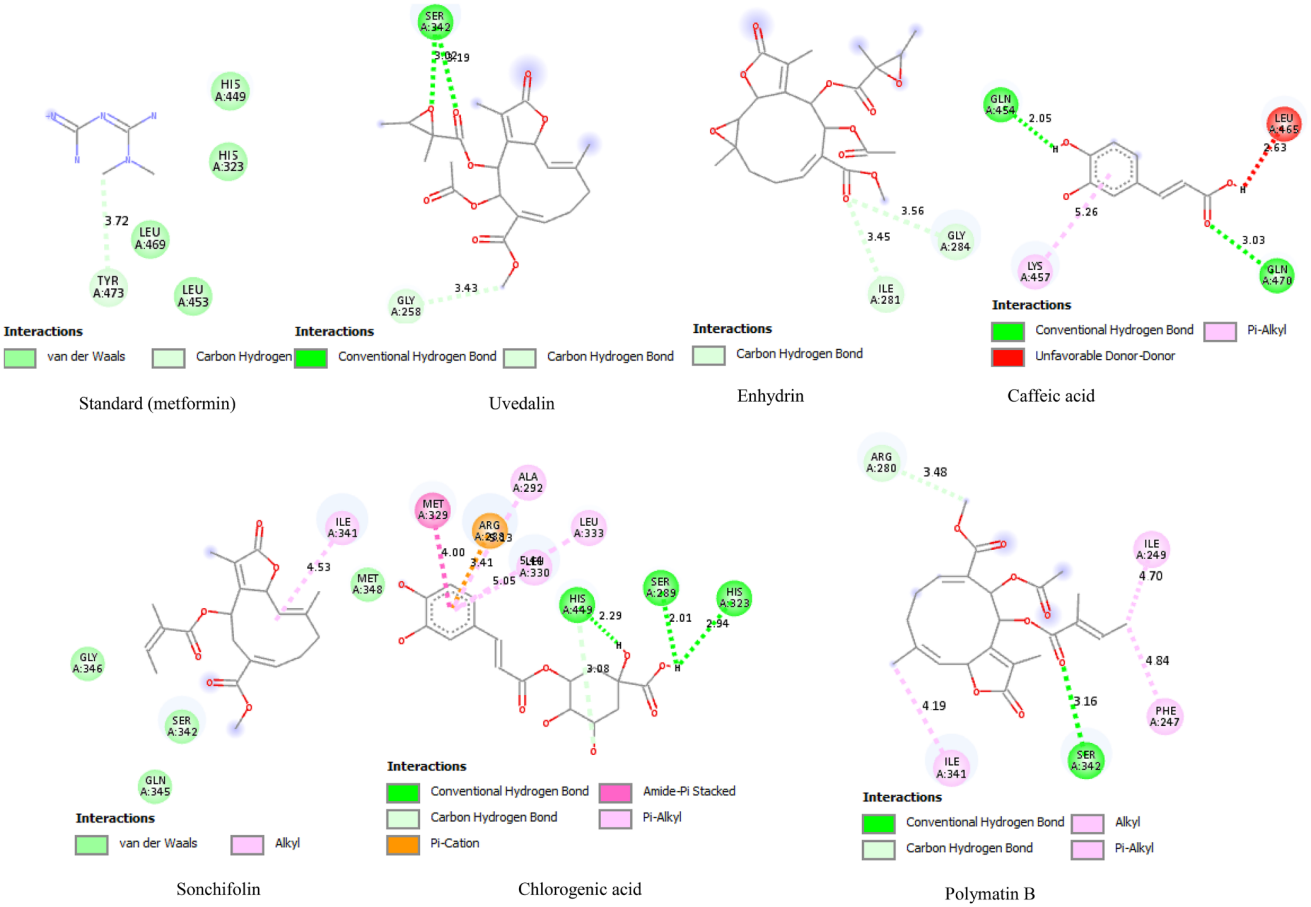
Interaction mode between the peroxisome proliferator activated receptor gamma (PPAR‐γ) (3G9E) catalytic site and phytoconstituents from leaves of *Smallanthus sonchifolius*.

All the phytoconstituents showed highest binding affinity ranging from −6.6 to −7.5 and −6 to −8.2 kcal/mol against alpha‐amylase and PPAR‐γ, respectively, compared to standard voglibose (−5.5 kcal/mol) and metformin (−4.5 kcal/mol) as shown in Figures 5 and 6 and Table 7.

**TABLE 7:** Binding energies (kcal/mol) of the phytoconstituents from *Smallanthus sonchifolius* against alpha‐amylase and PPAR‐γ.

Ligands	Alpha‐amylase (4 W93)		PPAR‐γ (3G9E)	
	Docking score	Interaction residue	Docking score	Interacting residue
Voglibose	−5.5	ASP 197, ASP 300, GLU 233, HIS 305	NA	NA
Metformin	NA	NA	−4.5	TRY 473
Uvedalin	−7.2	GLN 63, TRP 59	−6.9	GLY 258, SER 342
Enhydrin	−7.3	HIS 299, HIS 305	−6.8	GLY 284, ILE 281
Caffeic acid	−6.6	ASP 197, GLU 233	−6	GLN 354, GLN 470, LYS 457
Chlorogenic acid	−7.5	ASP 197, ASP 300, ARG 195, GLU 233, GLN 63, HIS 299, HIS 305, TRP 59	−7.2	ALA 292, ARG 288, HIS 449, LEU 330, LEU 333, LYS 267, MET 329, SER 289
Sonchifolin	−7.1	HIS 201	−7.1	ILE 341
Polymatin B	−7.3	ARG 195, GLU 233, HIS 305	−8.2	ARG 280, ILE 249, ILE 341, PHE 247, SER 342

*Note:* Docking score: kcal/mol.

Abbreviation: NA, not applicable.

## 4 Discussion

Natural products are recognized as the most important resource for the development of novel drugs in the pharmaceutical sector. Bioactive plant constituents have been used as substitutes for drug candidates and medicines for the treatment of different human diseases. In the present study, *S. sonchifolius* was selected for in vitro and in vivo studies based on its ethnomedicinal and traditional uses, as well as limited scientific exploration.

The aqueous and ethanolic extracts of this plant leaf revealed the presence of various chemical constituents, such as alkaloids, flavonoids, carbohydrates, phenols, and tannins, which may exhibit various pharmacological actions. Glycosides were not detected in any extract using the modified Brontrager test. Plant secondary metabolites showing antioxidant activity due to phenol and flavonoid content revealed a positive relationship with oral hypoglycemic agents (Hasan et al. 2017).

In the current study, water as a solvent for the extraction of *S. sonchifolius* leaf accounted for a higher extraction yield because of its high polarity and ability to solubilize hydrophilic phytoconstituents. Likewise, with regard to TPC as the standard gallic acid equivalent, the water extract of *S. sonchifolius* leaf possesses a high concentration of phenol (i.e., 88.46 ± 2.13 mg GAE/g dry extract). Moreover, aluminum chloride‐based spectrophotometric analysis for the estimation of flavonoid content revealed a prominent concentration of flavonoid in the water extract (i.e., 424.12 ± 2.86 mg QE/g dry extract weight). This demonstrates that the solvent system plays a major role in the solubility of some chemical constituents. It can be confirmed that solvents with higher polarity are more efficient for extracting phenolic compounds from plant parts than less polar solvents (Galanakis et al. 2013). This result is in agreement with that of a previous study (Russo et al. 2015a, 2015b).

In the DPPH assay, both water and ethanol extracts showed concentration‐dependent radical scavenging activity. Among them, water extracts of *S. sonchifolius leaf* (IC_50_ value of 47.64 μg/mL) showed strong radical scavenging activity compared to the extreme activity of standard ascorbic acid (IC_50_ value of 3.56 μg/mL). In this study, the IC_50_ value was found to be lower with an increase in both phenolic and flavonoid contents. The antioxidant activity of plant samples may be due to the presence of these chemical constituents (Yadav and Agrawal 2011). Valentova et al. (2003) reported that S. sonchifolius leaf extract shows potent antioxidant activity (IC_50_ value 24.3 ± 2.7 μg/mL) with a high percentage of inhibition of DPPH, which correlates with the present study that reveals the strong free radical scavenging properties of water and ethanol extracts of *S. sonchifolius* (Valentova et al. 2003).

Plants containing phenolic and flavonoid compounds exhibit free radical scavenging activity in living organisms (Aklima, Mojumder, and Sikdar 2014). Phenolic and flavonoid compounds are omnipresent groups of plant metabolites that exhibit pharmacological activities, such as antioxidant, antimicrobial, anti‐inflammatory, antidiabetic, antimutagenic, anticarcinogenic, and hepatoprotective activities (Sindhu et al. 2021; Pandey et al. 2024). Antioxidant type of compound mainly comes from plants in the form of secondary metabolite such as phenolic compounds (flavonoids, phenolic acids, and tocopherols). (Pandey et al. 2023). Phenolic compounds have antioxidant potential because they can scavenge free radicals and reactive oxygen species, such as superoxide free radicals, singlet oxygen, and hydroxyl radicals (Mishra, Kumar, and Pandey 2013). Flavonoids contain different functional hydroxyl groups that mediate their antioxidant activity by scavenging harmful free radicals and chelating metal ions to avoid harmful radical formation, which injures important biomolecules. Lipid peroxidation is the most common consequence of oxidative stress. Flavonoids play a major role in lipid peroxidation against oxidative damage via different routes (Kumar and Pandey 2012).

In the present study, the in vitro antidiabetic activity of both leaf extracts was evaluated via alpha‐amylase inhibition assay with molecular docking. Both plant extracts showed dose‐dependent inhibition of alpha‐amylase. The extract of *S. sonchifolius* (IC_50_ value 0.136) showed potent alpha‐amylase in alpha‐amylase activity against the alpha‐amylase enzyme in comparison with the standard voglibose (IC_50_ value of 0.126 mg/mL). As these extracts contain flavonoids, terpenoids, and saponins, due to the presence of these phytoconstituents it shows the alpha‐amylase inhibition activity (Uddin et al. 2014). Flavonoids, epicatechin, and epigallocatechin gallate are considered promising hypoglycemic agents (Gulati, Harding, and Palombo 2012). This herbal plant suggests a smart therapeutic advance for alpha‐amylase inhibition. Daily foods contain vegetables with high‐level alpha‐amylases, which is one of the best strategies for managing postprandial glucose levels (Gulati, Harding, and Palombo 2012). These phytochemicals are assumed to inhibit ch hydrolysis to smaller oligosaccharides, su, such as maltose and maltotriose by the enzyme alpha‐amylase. These smaller oligosaccharides are further acted upon by alpha‐glucosidase, which is converted to glucose and transported to the bloodstream by the hepatic portal vein. Inhibitions of the hydrolysis step of starch by alpha‐amylase therefore lower blood glucose levels. Therefore, this study aimed to determine the phytoconstituents of selected plant samples that inhibit these hydrolysis steps (Zinjarde, Bhargava, and Kumar 2011). Russo et al. (2015a, 2015b) reported that *S. sonchifolius* (yacon) leaf methanol extract showed strong inhibitors of alpha‐amylase (IC_50_ value 0.26 ± 0.02 mg/mL) that correlates with the present study that reveals strong alpha‐amylase inhibition activity of ethanolic extract of *S. sonchifolius* leaf. The alpha‐amylase inhibitory activity of *S. sonchifolius* may be attributed to caffeic and chlorogenic acids. These phenolic compounds are generally recognized for their antioxidant activity (Genta et al. 2010a, 2010b).

Among the solvent extracts of *S. sonchifolius* leaf, the ethanol extract was selected for an in vivo hypoglycemic activity study owing to its potent alpha‐amylase inhibitory activity. The in vivo hypoglycemic activity of *S. sonchifolius* leaf extract was investigated in normal mice and glucose hyperglycemic mice at three different doses (500, 250, and 100 mg/kg), and standard metformin (100 mg/kg) was used as a positive control. In normal mice, glucose levels in blood were increased immediately after administration of a different dosage of *S. sonchifolius* ethanolic extract, and at 60 and 120 min, *S. sonchifolius* (500 mg/kg) showed significant decreases in blood glucose levels (10.20% and 17.99%, respectively), which is comparable with metformin (100 mg/kg), given as a positive control, which showed decreased blood glucose levels by 26.14% and 31.86% at 60 and 120 min, respectively. The immediate increase in blood glucose levels in mice after administration of plant extracts may be due to the presence of reducing sugar content or as a physiological phenomenon (Khatri et al. 2018). The decrease in blood glucose levels at 60 and 120 min may be due to the presence of phytoconstituents that contribute to the hypoglycemic effects. The oral glucose tolerance test revealed that the ethanolic extract of *S. sonchifolius* decreased the blood glucose level by 5.16% after 60 min of glucose loading and was even lower at the end of 120 min (15.74%). This might be because *S. sonchifolius* improved glucose consumption, so the glucose level in blood was considerably diminished in glucose‐loaded mice. Genta et al. (2010a, 2010b) reported that the antihyperglycemic activity of *S. sonchifolius* might be due to phenolic compounds, caffeic and chlorogenic acids. Caffeic acid inhibits blood glucose levels, and chlorogenic acid improves glucose tolerance and insulin resistance in rats through an antioxidant mechanism. A significant decrease in blood glucose levels could be due to the presence of phytochemical constituents, such as anthraquinone, steroids, saponins, reducing sugars, tannins, and flavonoids. Several studies have suggested that flavonoids, steroids, terpenoids, and phenolic acids are hypoglycemic phytochemicals. Chen et al. (2008) reported that saponins in the root of *Panax notoginseng* showed significant antidiabetic effects in diabetic mice (Chen et al. 2008). Some studies have shown that plants with flavonoid compounds with alpha‐amylase inhibitory activity, enhanced glucose metabolism, and enhanced insulin secretion showed hypoglycemic activity (Shu et al. 2009).

Molecular docking plays a crucial role in predicting the binding site, affinity, and mode of interaction of drugs and chemicals with proteins, aiding a deeper understanding of the binding mechanism, and facilitating the design and discovery of novel therapeutic compounds (Das et al. 2024; Dilshad et al. 2024). The docking methodology was first validated using docking and re‐docking approaches. When the redocked ligand was overlaid with an earlier ligand, an RMSD value of 0.127 Å (< 2) was obtained, indicating that the docking process was validated. The docking was visualized in a 3D format, providing insights into the binding site, nature of bonding, bond length, and binding energy expressed in kcal/mol.

Alpha‐amylase is a crucial enzyme present in the salivary and pancreatic secretions. This endogenous enzyme is responsible for the digestion of dietary carbohydrates, such as starch, into maltose, which is responsible for increasing postprandial sugar levels in the blood via cleavage to glucose by alpha‐glucosidase in the intestine (Nasir et al. 2020; Sai et al. 2019); therefore, it is considered a potent target for novel antidiabetic drug development (Williams et al. 2012, 2015). Acarbose and voglibose are standard antidiabetic drugs that inhibit alpha‐amylase and glucosidase, thereby reducing postprandial blood sugar levels (Nasir et al. 2020). However, the adverse effects of gastrointestinal medications, including gastrointestinal pain, flatulence, and diarrhea, limit their therapeutic indications and patient compliance (Williams et al. 2015), which necessitates the development of new agents. Phytoconstituents have become a popular insight into controlling postprandial hyperglycemia by inhibiting alpha‐amylase (Nasir et al. 2020). With reference to the significant in vitro inhibitory activity of alpha‐amylase in *S. sonchifolius* extracts, we opted to forecast the *in silico* antibacterial properties of phytoconstituents against alpha‐amylase (Dilshad et al. 2024; Nwude, Osamudiamen, and Enessy 2024). All the phytoconstituents from *S. sonchifolius* were observed to bind with several amino residues via different bonds with a prominent affinity compared to standard voglibose, which satisfies the current in vitro studies and also supports several previous studies revealing the glucose‐lowering properties of leaf extract from *S. sonchifolius* (Genta et al. 2010a, 2010b; Russo et al. 2015a, 2015b) and alpha‐amylase inhibitory activity of its phytoconstituents enhydrin, uvedalin (Genta et al. 2010a, 2010b), caffeic acid (Mbagwu et al. 2022), and chlorogenic acid (Nyambe‐Silavwe and Williamson 2018). Specifically, chlorogenic acid were found to interact with all catalytic site amino acid of alpha‐amylase including ASP 197, ASP 300, and GLU 233 (Williams et al. 2012, 2015) via strong hydrogen bond (Figure 5) resulting the highest binding energy of −7.5 kcal/mol, representing a strong and perfect cholinergic acid‐alpha‐amylase complex and may provide precious lead structures in developing a new antidiabetic agents.

Peroxisome proliferator‐activated receptor (PPAR‐γ) is a nuclear receptor that plays a crucial role in the cellular ATP balance (Nolte et al. 1998). Biological conditions that inhibit ATP production, such as metabolic stress (hypoxia and low glucose level) and increased ATP consumption (exercise, cell growth, and differentiation phase), lead to an increase in the AMP:ATP ratio, thereby activating PPAR‐γ (Handa et al. 2011). PPAR‐γ enhances the sensitivity of cells to insulin (Nolte et al. 1998) and upregulates the genes responsible for glucose and lipid metabolism (Ali et al. 2024). Therefore, it is a promising pathway for the discovery of novel drugs against diabetes mellitus (Handa et al. 2011). Phytoconstituents from *S. sonchifolius* were observed to bind with several amino residues via different bonds with a prominent affinity compared to standard metformin, which verifies the current in vivo results and supports several previous studies revealing the antidiabetic effects of *S. sonchifolius* leaf extract (Genta et al. 2010a, 2010b; Russo et al. 2015a, 2015b). Especially, chlorogenic acid and polymatin B were seen interacting with maximum catalytic site amino acid of PPAR‐γ (Nolte et al. 1998), including HIS 449, SER 289, ARG 288 and LEU 330, and ILE 341 (Figure 6), respectively, via strong hydrogen bond resulting the maximum binding energy of −7.2 and − 8.2 kcal/m, representing a strong ligand‐protein complex and provide a precious drug candidates for diabetes.

The present study of *S. sonchifolius* revealed the existence of higher phenolic and flavonoid compounds together with potent antioxidant and alpha‐amylase activities and significant hypoglycemic activity. Hypoglycemic activity of this *S. sonchifolius* crude extract was also showed by the invitro activity which is also supported by the in vitro molecular docking studies, but to emphasize this future direction and the need for further research on individual phytoconstituents to elucidate the precise mechanisms involved. This could be a potential candidate for the development of novel oral hypoglycemic agents.

## 5 Conclusions

In summary, both ethanol and water extracts of *S. sonchifolius* leaf showed high content of phenolic and flavonoid compounds, which demonstrated noteworthy antioxidant activity. This study suggests that the ethanol extract of *S. sonchifolius* leaf showed high alpha‐amylase inhibitory activity, which could be responsible for its hypoglycemic effect. Additionally, the hypoglycemic action of *S. sonchifolius*, which inhibits the alpha‐amylase enzyme, is supported by molecular docking studies. Strong affinities of −8.2 and −7.2 kcal/mol for PPAR‐γ (3G9E) were found for polymatin B and chlorogenic acid, respectively. It is interesting to note that in alpha‐amylase (4 W93), polymatin B and chlorogenic acid both show the maximum binding affinities of −7.3 and −7.5 kcal/mol, respectively. Therefore, further studies isolating individual compounds such as chlorogenic acid and polymatin B are necessary to definitively confirm their effects on antidiabetic activity. Ultimately, our work clarifies a fantastic route for more clinical research and scientific confirmation, and investigation to identify the curative potential of this therapeutic plant for glycemic management in diabetic subjects and validate their method of action against diabetes.

## Author Contributions

**Prakash Rawal:** conceptualization (equal), data curation (equal), formal analysis (equal), investigation (equal), methodology (equal), project administration (equal), writing – original draft (equal). **Bipindra Pandey:** conceptualization (equal), data curation (equal), formal analysis (equal), investigation (equal), methodology (equal), software (lead), validation (equal), visualization (lead), writing – original draft (supporting), writing – review and editing (lead). **Ram Kishor Yadav:** data curation (supporting), investigation (supporting), methodology (supporting), software (supporting), validation (supporting), visualization (supporting), writing – review and editing (supporting). **Sushil Panta:** conceptualization (lead), investigation (equal), methodology (equal), project administration (lead), resources (lead), supervision (lead), validation (lead), writing – review and editing (lead).

## Ethics Statement

All experimental animals were handled according to the widely accepted guidelines for laboratory handling and the use of animals released by the National Institutes of Health (NIH, 2011). Prior to starting the animal experiments, approval was obtained from the Institutional Review Committee (Ref. No. 86/076/077) from Pokhara University, Pokhara, Nepal.

## Consent

The authors have nothing to report.

## Conflicts of Interest

The authors declare no conflicts of interest.

## Data Availability

All relevant data are in the paper, and any query regarding the findings of this study can be obtained from the corresponding author upon request.
